# Intimate partner violence and postpartum healthcare access in Kenya: a cross-sectional study

**DOI:** 10.1186/s12884-024-06342-0

**Published:** 2024-02-26

**Authors:** Rebecca Woofter, John Mboya, Ginger Golub, May Sudhinaraset

**Affiliations:** 1 Fielding School of Public Health, Department of Community Health Sciences, University of California Los Angeles, 650 Charles E. Young Dr. S, Los Angeles, CA 90095 USA; 2 Innovations for Poverty Action, New York, USA

**Keywords:** Intimate Partner violence, Pregnancy, Postpartum, Family Planning, Healthcare Access, Kenya

## Abstract

**Background:**

Intimate partner violence (IPV) impacts physical health, mental health, and healthcare use. IPV during pregnancy, in particular, is associated with lower rates of antenatal care, but no studies have assessed the association between IPV and postpartum healthcare. This study aims to examine the link between IPV (emotional, physical, and sexual) and two outcomes: postpartum healthcare use and access to family planning.

**Methods:**

This study uses data from a cross-sectional survey of 859 women in Nairobi and Kiambu counties in Kenya who gave birth during the COVID-19 pandemic in 2020.

**Results:**

In this sample, 36% of women reported ever experiencing IPV. Of those, 33% indicated the frequency of IPV stayed the same or increased during COVID-19. Nearly 17% of women avoided postpartum healthcare and 10% experienced issues accessing family planning. Those who experienced any form of IPV during pregnancy had approximately twice the odds of avoiding postpartum healthcare compared to those who did not experience any form of IPV. Compared to those who did not experience IPV during pregnancy, experiencing sexual IPV was associated with 2.25 times higher odds of reporting issues accessing family planning. Additionally, reporting fair or poor self-rated health was associated with both avoiding postpartum healthcare and reporting issues accessing family planning. Experiencing food insecurity was also associated with avoiding postpartum healthcare.

**Conclusions:**

To our knowledge, this is the first study to establish the link between IPV during pregnancy and postpartum healthcare access. During COVID-19 in Kenya, postpartum women who had experienced IPV were at increased risk of disengagement with healthcare services. Women should be screened for IPV during pregnancy and postpartum in order to better support their healthcare needs. In times of crisis, such as pandemics, policymakers and healthcare providers must address barriers to healthcare for postpartum women.

## Background

Among the many consequences of the COVID-19 pandemic, concern for increased violence against women and lack of access to support resources has been a priority for public health and medical professionals [1]. During previous infectious disease outbreaks, such as Ebola and Zika, instances of intimate partner violence (IPV) increased globally, leading many to believe a similar pattern would emerge with COVID-19 [2]. Lockdown and quarantine procedures to mitigate the spread of disease could result in isolation, which, in combination with economic and psychological stressors, may put women at risk of experiencing violence [3]. While empirical studies of the burden of IPV during the COVID-19 pandemic are limited, a rapid literature review of global articles found dramatic increases in calls to violence helplines, with some reduction in use of in-person services [4]. In Kenya, the increase in violence against women and girls during the COVID-19 pandemic led the government to call for investigations into the reasons for the rise in violence and its impact on women [5]. A report from July 2020 with survivors of violence during the pandemic highlights the connection between the emergency lockdown measures enacted in March 2020, resulting socioeconomic precarity, and abuses by police enforcing lockdown orders with the increased level of violence [5].

IPV is one type of violence that is of particular concern due to its negative implications. IPV refers to abuse of a physical, sexual, or emotional nature that is perpetrated by an individual on their intimate partner, and is associated with poor mental and physical health [6]. Pregnancy is a particularly vulnerable period for IPV, with heightened household tension and increased need for resources [6]. IPV that occurs during pregnancy is associated with lower attendance at antenatal care visits during pregnancy, resulting in harmful infant outcomes such as miscarriage and low birth weight [6]. Notably, IPV can decrease healthcare seeking either by decreasing desire to engage with healthcare due to mental health effects of IPV, or by coercion inflicted by a partner leading to low levels of personal autonomy [7, 8]. Indeed, three recent systematic and scoping reviews found that IPV was consistently linked with later initiation of antenatal care, fewer overall antenatal care visits, and lower likelihood of delivery with a trained provider [8–10]. However, no studies to date have examined the association between IPV during pregnancy and access to healthcare in the postpartum period.

Postpartum healthcare, focused on the mother in the weeks after delivery, is vital for maternal health. The World Health Organization (WHO) recommends at least three healthcare visits in the weeks after delivery for both infant and maternal health [11]. During these visits, postpartum women should be assessed for mental health and signs of postpartum depression, overall wellbeing, and physical recovery from delivery [11]. Most maternal deaths occur in the postpartum period, and timely access to postpartum healthcare is vital for preventing maternal mortality [11]. At 342 maternal deaths per 100,000 live births, Kenya has the 31st highest maternal mortality ratio in the world [12]. However, less than half of postpartum women in Kenya receive any postpartum services [13].

In addition to preventing maternal mortality, postpartum healthcare provides an avenue for women to receive family planning. Indeed, accessing postpartum healthcare is associated with use of postpartum family planning [14]. Initiation of family planning in the weeks after delivery ensures that women can increase spacing between births and ultimately decreases maternal mortality [15]. Though not limited to the pregnancy period, a systematic review of twelve studies found that women reporting IPV had over 50% lower odds of using family planning compared to those not reporting IPV [16].

Despite the clear evidence on the negative impacts of IPV during pregnancy, including delayed and/or reduced levels of antenatal care, gaps in the literature remain on the association between IPV and postpartum healthcare access. This study aims to address that gap by assessing the relationship between IPV during pregnancy and two postpartum healthcare outcomes: avoiding postpartum healthcare and experiencing issues accessing family planning after delivery.

## Methods

### Recruitment & data collection

This study includes a sample of 1,135 women enrolled in the iDELIVER (Identifying effective DELIVERy models during COVID-19) study of health-seeking behaviors among postpartum women during the COVID-19 pandemic. Eligible women included those aged 15–49 years who had a singleton birth since COVID-19 restrictions were instated by the government of Kenya (March 16th, 2020), resided within the catchment areas surrounding six health facilities in Nairobi and Kiambu counties, and possessed a functional mobile phone to allow for remote surveying. Women were identified and recruited by community leaders and community health volunteers. A total of 2,011 women were identified and contacted, of whom 1,135 were successfully reached and consented to participate.

Data were collected between September and November 2020 by nine experienced female enumerators who completed an intensive five-day training on the study procedures, consent process, and questionnaire. Each woman identified for participation was contacted up to nine times, varying the day and time of attempt. Upon reaching a participant, the enumerators described the study and obtained verbal informed consent for participation and audio recording of the conversation before completing the survey. The consent process and survey were conducted verbally with the trained enumerators in either Kiswahili and English, whichever the participant preferred. Women who consented and completed any part of the survey received the equivalent of approximately $1 in mobile phone airtime in appreciation of their time and participation.

### Measures

This analysis considers two binary outcomes of interest related to postpartum healthcare access. The first, *avoiding postpartum healthcare*, was asked in the survey as: “Since your delivery, have you needed care but avoided using or were otherwise unable to use health services or visit health care providers?” Women who indicated that they avoided postpartum healthcare were asked two follow-up questions: “What services have you needed but avoided or have been unable to access?” and, “What were the reasons you avoided or were unable to attend these services?” The second outcome, *issues accessing family planning*, was asked in the survey as: “Have you experienced any issues when trying to receive or obtain a family planning method since COVID-19 (mid-March)?” Those who reported experiencing these issues were asked a follow-up question: “What issues have you experienced?” Although not part of the quantitative analyses, the follow-up questions were used to better understand the nature and reasons for lack of access to postpartum healthcare.

The exposure of interest in this analysis is experience of intimate partner violence. Intimate partner violence was measured using five selected items from the domestic violence module of the Demographic and Health Survey [17]. These questions were asked of participants who were currently or previously partnered. They were asked as: “Does your partner ever say or do something to humiliate you in front of others?”; “Does your partner ever threaten you or someone close to you with harm?”; “Does your partner ever push you, shake you, or throw something at you?”; “Does your partner ever slap you or twist your arm?”; and, “Does your partner ever physically force you to have sexual intercourse with him even when you did not want to?” To gauge any changes in IPV experiences during the COVID-19 pandemic, an additional question was asked of those who reported at least one IPV experience in the previous set of questions: “In general, would you say that these types of behaviors by your partner have increased, decreased, or not changed since March?,” with options of increased, decreased, no change, or not applicable (no longer with partner). These six IPV variables were recoded into four exposure variables. The variables were: reporting any form of IPV, reporting physical IPV, reporting emotional IPV, and reporting sexual IPV. In order to categorize respondents based on when they experienced IPV relative to COVID-19, respondents were coded for each of the types of IPV as never experiencing it (y = 0), experiencing it but it decreased during COVID-19 or is no longer applicable (y = 1), and experiencing it at the same or an increased rate during COVID-19 (y = 2). Given that each participant gave birth since the beginning of the pandemic, those who report the frequency of IPV staying the same or increasing during the pandemic can reasonably be assumed to have experienced IPV during their pregnancy.

Final models were adjusted for demographic covariates including age (ranging from 16–49) and parity (1, 2, 3, 4+), socioeconomic status covariates including level of completed education (none/some primary, primary/some secondary, secondary, college/university), current employment status (employed or unemployed), and food insecurity in the past month, and an indicator of overall health, self-rated health (excellent/very good/good, fair/poor/very poor), Food insecurity was measured with six items from the Household Food Insecurity Access Scale [18]. These items asked about experiences of food insecurity in the last four weeks and were combined into one continuous composite score, where a higher score indicates more experiences of food insecurity (ranging from 0 to 6).

These covariates were selected as they are each theorized to be associated with both IPV and postpartum healthcare access, and thus could confound this relationship. Literature suggests that those of lower socioeconomic status are at greater risk of IPV [19, 20]. Those with lower socioeconomic status may experience more household tension due to scarce resources and be at higher risk of IPV, and may experience barriers to accessing healthcare based on lack of funds. While some studies use household income as measures of socioeconomic status, food insecurity represents a useful element of socioeconomic status, as it demonstrates the ability to afford and access sufficient food. This is a dynamic measure of socioeconomic status that changed drastically in Kenya during the COVID-19 pandemic, as estimates suggest food insecurity increased by nearly 40% in Kenya [21]. Self-rated health was included in order to control for differences in physical health among the women, and thus differences in the level of need for postpartum healthcare. Prior studies have noted that women with chronic conditions or complications during pregnancy or delivery tend to have higher rates of postpartum care use compared to those without these health complications [22].

### Analyses

Analyses began with univariate examination of postpartum healthcare access, intimate partner violence, and sociodemographic and health characteristics. Next, bivariate associations between each form of IPV and the two outcomes were determined using chi-squared tests. Finally, four multivariable logistic regression models were completed for each outcome, with separate models for each form of intimate partner violence (any, emotional, physical, sexual). All analyses were conducted using STATA SE/16.

## Results

### Sample demographics

Of the 1,135 women in the survey, those who indicated they were currently or ever partnered (married, in a relationship, divorced, or widowed) were asked about experiences of IPV, resulting in an analytic sample of 861 for this analysis. Two women did not respond to some of the IPV questions and were thus excluded, leaving a final sample of 859.

**Table 1:** Demographic characteristics (*N* = 859)

Variable	N (%) or Mean (SD)
**Postpartum Healthcare**	
*Did not avoid healthcare*	715 (83.2)
*Avoided healthcare*	144 (16.8)
**Family Planning Access**	
*Did not experience access issues*	777 (90.5)
*Experienced access issues*	82 (9.6)
**Any Form of IPV**	
*Never occurred*	549 (63.9)
*Stayed the same or increased during COVID-19*	209 (24.3)
*Decreased or did not occur during COVID-19*	101 (11.8)
**Emotional IPV**	
*Never occurred*	716 (83.4)
*Stayed the same or increased during COVID-19*	88 (10.2)
*Decreased or did not occur during COVID-19*	55 (6.4)
**Physical IPV**	
*Never occurred*	592 (68.9)
*Stayed the same or increased during COVID-19*	178 (20.7)
*Decreased or did not occur during COVID-19*	89 (10.4)
**Sexual IPV**	
*Never occurred*	727 (84.6)
*Stayed the same or increased during COVID-19*	83 (9.7)
*Decreased or did not occur during COVID-19*	49 (5.7)
**Marital Status**	
*Married or partnered*	770 (89.6)
*Widowed or divorced*	89 (10.4)
**Age**	
*< 25*	276 (32.1)
*25–29*	278 (32.4)
*30–34*	199 (23.2)
*35+*	106 (12.3)
**Parity**	
*1*	198 (23.1)
*2*	280 (32.6)
*3*	216 (25.2)
*4+*	165 (19.2)
**Education**	
*No school or some primary school*	97 (11.3)
*Primary or some secondary school*	363 (42.3)
*Secondary school*	305 (35.5)
*College/University*	94 (10.9)
**Employment Status**	
*Not employed*	695 (80.9)
*Employed*	164 (19.1)
**Food Insecurity (#, range 0–6)**	3.7 (1.8)
**Self-rated Health**	
*Excellent, very good, or good*	559 (65.1)
*Fair, poor, or very poor*	300 (34.9)

Table 1 demonstrates the percent of women in the sample who experienced each outcome of interest. Nearly 17% of women indicated they had avoided postpartum healthcare. Those who had avoided postpartum healthcare were then asked what type(s) of healthcare they needed but avoided. The two most common types of care avoided were emergency care (49%) and routine care or check-ups, including postnatal care (23%). Women also reported a variety of reasons why they avoided postpartum healthcare. Most commonly, about 40% of women reported they could not afford healthcare due to COVID-19 and 40% reported that they were scared of going out due to the risk of contracting COVID-19.

On the second outcome, about 10% of women indicated that they experienced issues accessing family planning during COVID-19. When asked the reasons for these issues, nearly 49% of women indicated that the facility or pharmacy did not have a family planning method available and nearly 20% reported that they were unable to afford a family planning method. Notably, among those who avoided postpartum care, 20% also reported experiencing issues accessing family planning.

Table 1 also shows the sociodemographic and health characteristics of the sample. Approximately 90% of the women in the sample were currently married or partnered, while 10% were widowed or divorced. The sample ranged in age from 16 to 49, with about two-thirds under the age of 30. Over 19% had three or more previous children, up to a maximum of six previous children. 42% of women had primary or some secondary school, while 36% had finished secondary school. While 22% of women were not employed before the COVID-19 pandemic, over 58% became unemployed during the pandemic for a total of 81% unemployed. For nearly one-quarter of the sample, the child they delivered during COVID-19 was their first child. About one-third already had one previous child, and one-quarter already had two previous children. The average number of food insecurity experiences was 3.7, and over 19% of the sample reported all six experiences. Less than 10% of the sample reported no experiences of food insecurity in the past month. Over one-third of women rated their health as fair, poor, or very poor.

**Fig. 1:**
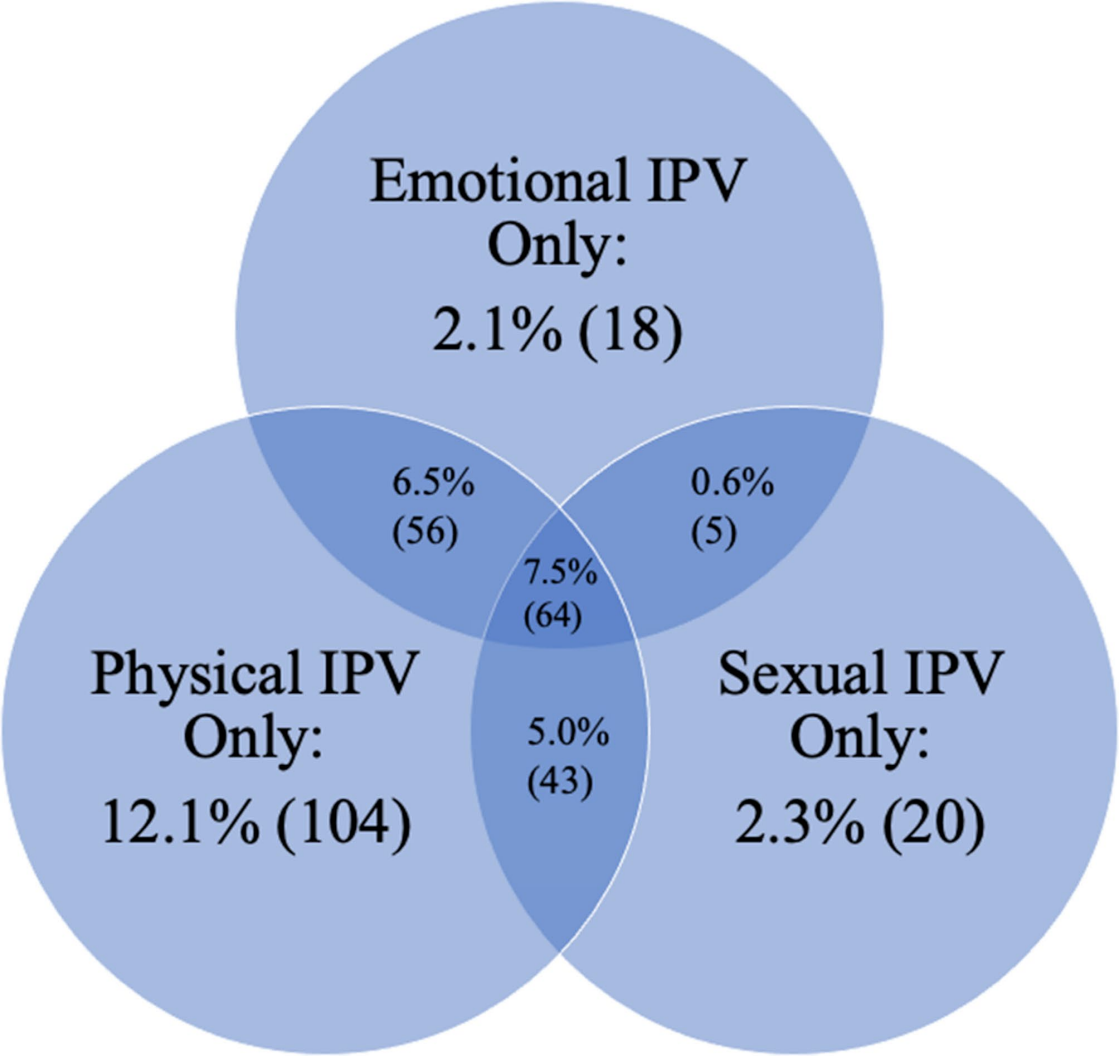
Prevalence of each form of intimate partner violence ever experienced among postpartum women (*N* = 859)

Figure 1 shows the prevalence of intimate partner violence reported in this sample. Over 36% of the women reported at least one form of intimate partner violence ever occurring. Physical intimate partner violence was most commonly reported of the forms of IPV at over 31% of the sample, followed by emotional and sexual intimate partner violence, reported by approximately 17% and 15% of the sample, respectively. Notably, many women experienced more than one form of violence. Nearly 8% of the sample reported experiencing all three forms of violence. Furthermore, over 4% of women indicated they had experienced all five of the intimate partner violence indicators.

Table 1 displays experiences of intimate partner violence categorized by when they occurred. About 12% of the sample reported at least one form of violence that stayed the same or increased during COVID-19. Over 10% reported physical violence that stayed the same or increased during COVID-19, while around 6% of the sample reported emotional and sexual violence that stayed the same or increased during COVID-19, each. When looking only among those who reported ever experiencing IPV, 19% indicated that it increased during COVID-19, 50% indicated that it decreased, and 14% indicated no change in frequency.

### Bivariate analyses

**Table 2:** Bivariate associations of intimate partner violence with postpartum healthcare and access to family planning (*N* = 859)

	Avoided Postpartum Healthcare	Chi-squared ***P***-Value	Experienced Issues Accessing Family Planning	Chi-squared ***P***-Value
**Any Form of IPV**		0.004		0.001
*Never occurred*	77 (14.0)		40 (7.3)	
*Stayed the same or increased during COVID-19*	40 (19.1)		23 (11.0)	
*Decreased or did not occur during COVID-19*	27 (26.7)		19 (18.8)	
**Emotional IPV**		<0.001		0.017
*Never occurred*	101 (14.1)		61 (8.5)	
*Stayed the same or increased during COVID-19*	25 (28.4)		10 (11.4)	
*Decreased or did not occur during COVID-19*	18 (32.7)		11 (20.0)	
**Physical IPV**		0.009		0.192
*Never occurred*	86 (14.5)		51 (8.6)	
*Stayed the same or increased during COVID-19*	34 (19.1)		18 (10.1)	
*Decreased or did not occur during COVID-19*	24 (27.0)		13 (14.6)	
**Sexual IPV**		0.001		0.004
*Never occurred*	110 (15.1)		61 (8.4)	
*Stayed the same or increased during COVID-19*	17 (20.5)		10 (12.1)	
*Decreased or did not occur during COVID-19*	17 (34.7)		11 (22.5)	

Table 2 depicts bivariate analyses between each type of IPV and each of the two outcomes. Among those who reported any form of IPV that stayed the same or increased during COVID-19, 19% avoided postpartum healthcare and 11% experienced issues accessing family planning. Among those who reported emotional IPV that stayed the same or increased during COVID-19, 28% avoided postpartum healthcare and 11% experienced issues accessing family planning. Among those who reported physical IPV that stayed the same or increased during COVID-19, 19% avoided postpartum healthcare and 10% experienced issues accessing family planning. Among those who reported sexual IPV that stayed the same or increased during COVID-19, 21% avoided postpartum healthcare and 12% experienced issues accessing family planning. Chi-squared tests indicate that avoiding postpartum healthcare statistically significantly differed by each experience of IPV (*p* < 0.01). Experiencing issues accessing family planning statistically significantly differed by reports of any form of IPV, emotional IPV, and sexual IPV (*p* < 0.05), though not by reports of physical IPV.

### Multivariable analyses

**Table 3:** Odds of avoiding postpartum healthcare based on experiences of intimate partner violence with covariates (*N* = 859)

	Model 1: Any IPV aOR (95% CI)	Model 2: Emotional IPV aOR (95% CI)	Model 3: Physical IPV aOR (95% CI)	Model 4: Sexual IPV aOR (95% CI)
**Experience of IPV**				
*Never occurred*	Ref	Ref	Ref	Ref
*Decreased or did not occur during COVID-19*	1.12 (0.71, 1.77)	2.04 (1.19, 3.52)*	1.09 (0.68, 1.76)	1.09 (0.60, 2.00)
*Stayed the same or increased during COVID-19*	1.78 (1.03, 3.06)*	2.41 (1.27, 4.56)**	1.77 (1.01, 3.10)*	2.32 (1.19, 4.51)*
**Age**				
*< 25*	Ref	Ref	Ref	Ref
*25–29*	1.10 (0.64, 1.88)	1.07 (0.62, 1.83)	1.10 (0.64, 1.88)	1.12 (0.65, 1.91)
*30–34*	1.56 (0.83, 2.94)	1.50 (0.80, 2.81)	1.59 (0.84, 2.98)	1.61 (0.85, 3.03)
*35+*	1.61 (0.75, 3.44)	1.55 (0.72, 3.32)	1.62 (0.76, 3.47)	1.62 (0.76, 3.46)
**Education**				
*No school or some primary school*	Ref	Ref	Ref	Ref
*Primary or some secondary school*	0.80 (0.42, 1.52)	0.90 (0.47, 1.71)	0.81 (0.43, 1.53)	0.83 (0.44, 1.58)
*Secondary school*	1.46 (0.76, 2.82)	1.65 (0.85, 3.23)	1.47 (0.76, 2.83)	1.51 (0.87, 2.93)
*College or university*	0.88 (0.36, 2.16)	1.00 (0.40, 2.47)	0.89 (0.36, 2.19)	0.89 (0.36, 2.17)
**Employment Status**				
*Not employed*	Ref	Ref	Ref	Ref
*Employed*	1.00 (0.62, 1.63)	0.99 (0.61, 1.61)	1.00 (0.62, 1.62)	0.98 (0.60, 1.60)
**Parity**				
*1*	Ref	Ref	Ref	Ref
*2*	0.92 (0.52, 1.60)	0.95 (0.54, 1.67)	0.91 (0.52, 1.59)	0.91 (0.52, 1.59)
*3*	0.64 (0.32, 1.26)	0.65 (0.33, 1.29)	0.63 (0.32, 1.25)	0.64 0.32, 1.27)
*4+*	0.79 (0.37, 1.70)	0.79 (0.37, 1.70)	0.79 (0.37, 1.69)	0.79 (0.37, 1.70)
**Self-rated Health**				
*Excellent, very good, or good*	Ref	Ref	Ref	Ref
*Fair, poor, or very poor*	2.02 (1.38, 2.95)***	1.98 (1.35, 2.89)***	2.03 (1.39, 2.97)***	2.03 (1.39, 2.96)***
**Food Insecurity (#, range 0–6)**	1.22 (1.08, 1.37)**	1.20 (1.07, 1.36)**	1.22 (1.08, 1.38)**	1.21 (1.08, 1.37)**

Note: *p < 0.05, **p < 0.01, ***p < 0.001

Table 3 describes the four multivariable models conducted to estimate the odds of avoiding postpartum healthcare based on reporting each form of IPV. Compared to those who did not report any form of IPV, those who reported any form of IPV that stayed the same or increased during COVID-19 had 78% higher odds of avoiding postpartum healthcare (aOR 1.78, 95%CI: 1.03, 3.06). Compared to those who did not report emotional IPV, those who reported emotional IPV that stayed the same or increased during COVID-19 had 2.4 times higher odds of avoiding postpartum healthcare (aOR 2.41, 95%CI: 1.27, 4.56). Additionally, those who reported emotional IPV that decreased or did not occur during COVID-19 had twice the odds of avoiding postpartum healthcare (aOR 2.04, 95%CI: 1.19, 3.52). Compared to those who did not report physical IPV, those who reported physical IPV that stayed the same or increased during COVID-19 had 77% higher odds of avoiding postpartum healthcare (aOR 1.77, 95%CI: 1.01, 3.10). Compared to those who did not report sexual IPV, those who reported any form of IPV that stayed the same or increased during COVID-19 had 2.3 times higher odds of avoiding postpartum healthcare (aOR 2.32, 95%CI: 1.19, 4.51). All of these findings were statistically significant at *p* < 0.05 or p<0.01. Those who reported physical, sexual, or any form of IPV that decreased or did not occur during COVID-19 did not have odds of avoiding postpartum healthcare that statistically significantly differed from those who reported never experiencing IPV, respectively.

**Table 4:** Odds of experiencing issues accessing family planning during COVID-19 based on experiences of intimate partner violence with covariates (*N* = 859)

	Model 1: Any IPV aOR (95% CI)	Model 2: Emotional IPV aOR (95% CI)	Model 3: Physical IPV aOR (95% CI)	Model 4: Sexual IPV aOR (95% CI)
**Experience of IPV**				
*Never occurred*	Ref	Ref	Ref	Ref
*Decreased or did not occur during COVID-19*	1.23 (0.69, 2.22)	1.01 (0.47, 2.13)	0.87 (0.47, 1.62)	1.10 (0.52, 2.35)
*Stayed the same or increased during COVID-19*	2.25 (1.18, 4.29)*	1.96 (0.92, 4.19)	1.28 (0.64, 2.59)	2.26 (1.05, 4.88)*
**Age**				
*< 25*	Ref	Ref	Ref	Ref
*25–29*	0.38 (0.18, 0.81)*	0.38 (0.18, 0.79)*	0.38 (0.18, 0.81)*	0.39 (0.19, 0.82)*
*30–34*	0.70 (0.32, 1.54)	0.70 (0.32, 1.53)	0.71 (0.33, 1.56)	0.73 (0.33, 1.60)
*35+*	0.69 (0.27, 1.76)	0.66 (0.26, 1.67)	0.64 (0.25, 1.63)	0.68 (0.27, 1.72)
**Education**				
*No school or some primary school*	Ref	Ref	Ref	Ref
*Primary or some secondary school*	0.78 (0.38, 1.60)	0.81 (0.39, 1.67)	0.78 (0.38, 1.61)	0.81 (0.39, 1.66)
*Secondary school*	0.88 (0.40, 1.92)	0.90 (0.41, 1.98)	0.86 (0.40, 1.87)	0.90 (0.41, 1.97)
*College or university*	0.90 (0.31, 2.61)	0.93 (0.32, 2.69)	0.88 (0.30, 2.53)	0.90 (0.31, 2.58)
**Employment Status**				
*Not employed*	Ref	Ref	Ref	Ref
*Employed*	1.26 (0.71, 2.24)	1.28 (0.72, 2.27)	1.31 (0.74, 2.32)	1.25 (0.70, 2.22)
**Parity (#)**				
*1*	Ref	Ref	Ref	Ref
*2*	1.15 (0.54, 2.48)	1.17 (0.55, 2.49)	1.16 (0.55, 2.47)	1.15 (0.54, 2.46)
*3*	1.48 (0.60, 3.64)	1.60 (0.66, 3.91)	1.59 (0.65, 3.92)	1.54 (0.63, 3.78)
*4+*	2.26 (0.85, 6.03)	2.38 (0.89, 6.31)	2.45 (0.92, 6.52)	2.33 (0.88, 6.22)
**Self-rated Health**				
*Excellent, very good, or good*	Ref	Ref	Ref	Ref
*Fair, poor, or very poor*	2.77 (1.70, 4.51)***	2.85 (1.75, 4.64)***	2.90 (1.78, 4.71)***	2.83 (1.74, 4.60)***
**Food Insecurity (#, range 0–6)**	1.00 (0.86, 1.16)	1.02 (0.88, 1.18)	1.04 (0.90, 1.20)	1.01 (0.87, 1.17)

‘Note: *p < 0.05, **p < 0.01, ***p < 0.001

Table 4 describes the four multivariable models conducted to estimate the odds of experiencing issues accessing family planning based on reporting each form of IPV. Compared to those who did not report any form of IPV, those who reported any form of IPV that stayed the same or increased during COVID-19 had 2.3 times higher odds of experiencing issues accessing family planning (aOR 2.25, 95%CI: 1.18, 4.29). Compared to those who did not report sexual IPV, those who reported sexual IPV that stayed the same or increased during COVID-19 had 2.3 times higher odds of experiencing issues accessing family planning (aOR 2.26, 95%CI: 1.05, 4.88). These findings were both statistically significant at *p* < 0.05. Sexual IPV or any form of IPV that decreased or did not occur during COVID-19 did not have odds of experiencing issues accessing family planning that statistically significantly differed from those who reported never experiencing IPV.

Those who reported physical or emotional violence, regardless of change during COVID-19, did not have odds of experiencing issues accessing family planning that statistically significantly differed from those who reported never experiencing that type of IPV, respectively.

## Discussion

This paper is the first, to our knowledge, to examine the association between experiencing IPV during pregnancy and access to postpartum healthcare and family planning. This study found that any experience of IPV during pregnancy is statistically significantly associated with avoiding postpartum healthcare. While experiencing each form of IPV during pregnancy was independently associated with avoiding postpartum healthcare, experiencing emotional IPV was most strongly associated with avoiding postpartum healthcare. Experiencing any form of IPV during pregnancy, and specifically sexual IPV, was statistically significantly associated with experiencing issues accessing family planning during the study period. Additionally, poor self-rated health was associated with both avoiding postpartum healthcare and experiencing issues accessing family planning, while food insecurity was associated with avoiding postpartum healthcare only.

In this study, 36% of the sample had ever experienced a form of IPV. This is similar to national estimates from before the COVID-19 pandemic. In the 2014 Kenyan Demographic Health Survey, 32% of currently married or partnered women reported ever experiencing a form of IPV [23]. Another analysis of these data found that approximately 9% of women reported experiencing IPV during pregnancy in particular [19]. However, the prevalence of IPV in this study is lower than in other region-specific studies of pregnant and postpartum women within Kenya. This may be due to the present sample coming from the more urban counties of Nairobi and Kiambu, while other studies focused on more rural areas, which are known to have higher rates of IPV. One study of pregnant women in Kisumu found that 53% had ever experienced IPV, 52% had experienced IPV in the 12 months before pregnancy, and 37% had experienced IPV during the current pregnancy [24]. A study of pregnant women in West Pokot County found that 67% of women had experienced IPV during their current pregnancy [25]. One study of postpartum women in Eldoret found that 34% experienced a form of IPV during their most recent pregnancy and 36% experienced IPV before their most recent pregnancy [26]. Most of these studies found that emotional IPV was most common, followed by physical IPV, and finally sexual IPV. In contrast, in the current study, physical IPV was most common, followed by emotional IPV, and finally sexual IPV. One previous study also found this trend [19]. It could be that the heightened pandemic-related stressors manifested as physical violence during this period. It could also be related to measurement of IPV, as more questions captured physical violence than emotional or sexual violence in the present study.

As this study was cross-sectional, it cannot determine whether IPV overall changed before and during the COVID-19 pandemic. However, by asking participants whether their own experience of IPV increased since the beginning of the pandemic, it is possible to estimate this change at the individual level. Among those who ever experienced IPV, nearly one in five indicated the IPV increased during the pandemic and one-third indicated the IPV either increased or stayed the same during the pandemic. While few studies have examined changes in IPV during pregnancy in the COVID-19 context, one longitudinal cohort study in Ethiopia found a 50% increase in the prevalence of IPV during pregnancy, from 10 to 15% of their sample [27]. Though not specific to pregnancy, one study in Nairobi found that 36% of women reported increased household tension and 6% reported increased violence in the household in May of 2020 compared to April of 2020 [28]. Similar to the estimate in the present analysis, a prospective study in Nairobi found that about 28% of partnered adolescent girls and young women experienced IPV during the pandemic, with higher rates reported among those with lower levels of education and socioeconomic status [29]. Additionally, the authors determined that IPV was more commonly reported in the late pandemic (in 2021) than earlier in the pandemic, suggesting that trends in IPV may have changed over time within the pandemic [29]. Researchers should prioritize investigating changes in violence experienced by vulnerable populations, such as pregnant individuals, during emergencies like the COVID-19 pandemic.

Some studies suggest reasons why IPV may have increased during the COVID-19 pandemic. A study of community health workers in Kenya, Bangladesh, and Haiti found that triggers for IPV during COVID-19 included partners spending more time together at home, conflicts over household responsibilities including childcare, and increased substance use [30]. In their mixed-methods analysis, Decker and colleagues similarly found that increased time spent with partners, disputes over household tasks – and the resulting threats to gender relations – as well as financial stress contributed to increased IPV [29]. Over half of those surveyed in Kenya indicated they perceived increased levels of IPV during the pandemic compared to before the pandemic [30]. Further research is needed to understand the influences contributing to IPV during the COVID-19 pandemic.

While no available literature exists on the association between IPV and postpartum healthcare, some comparisons can be made with literature on the association between IPV and antenatal care and intrapartum healthcare. A scoping review of 16 studies from 10 low- and middle-income countries found consistent negative associations between IPV and initiation of antenatal care, total number of antenatal care visits, quality of antenatal care, and attendance at birth by a skilled provider [8]. In Kenya specifically, only two studies to date have examined IPV and perinatal healthcare use. An analysis of the 2008–2009 Kenyan Demographic and Health Survey examined IPV and antenatal care visits among women aged 15–24 with a child under the age of five [31]. They found that those who experienced IPV in the past year had lower odds of attending at least four antenatal visits for their most recent birth [31]. Notably, though, the antenatal care in this study may have preceded the experiences of IPV, as the sample included women who had a birth in the last five years but measured IPV in the last twelve months. Additionally, this study did not capture emotional IPV [31]. A second study by Goo and Harlow used the 2003 Kenyan Demographic and Health Survey to determine whether IPV was associated with delivering with a skilled provider at birth in the past year [32]. They included measures for physical, sexual, and emotional IPV [32]. Lifetime experience of emotional or physical IPV was associated with lower odds of having a skilled birth attendant [32]. The present study expands these earlier studies by demonstrating that physical, emotional, and sexual IPV are all associated with avoidance of postpartum healthcare.

Some of these studies suggest mechanisms by which IPV may be associated with lower levels of healthcare access during pregnancy and delivery. Within the studies reviewed by Metheny and Stephenson, the most common reason suggested for decreased use of antenatal care among pregnant women who experienced IPV was lack of personal autonomy [8]. For example, experiencing IPV may reduce women’s decision-making power and ability to attend healthcare visits. Some other studies concluded that experiencing IPV decreased desire to seek healthcare based on reduced psychosocial well-being [8]. Goo and Harlow suggest that IPV may serve as a proxy for gender power dynamics in which partners could exercise control over pregnant women and dictate their level of engagement with healthcare services [31]. Some studies in Kenya provide additional context for IPV in this setting. Memiah and colleagues indicate that cultural sanctioning of gender power imbalance or tolerance for IPV in Kenya may be related to IPV victims’ lack of autonomy in accessing healthcare [20]. Similarly, qualitative focus groups and interviews with pregnant women, their partners and male family members, and service providers in Nyanza, Kenya demonstrated that IPV was normalized in this setting [33]. Another qualitative study with pregnant women, their partners, and service providers in Kenya found community norms accepting IPV, and highlighted the increased risk of IPV when men were unable to meet gender expectations of providing financially for the family [34]. COVID-19 may have exacerbated this pathway, as many men lost their jobs or received decreased wages during the pandemic. Another analysis of the dataset used in the current study found that women who experienced job loss or reduction in the household due to COVID-19 faced higher rates of postpartum depression [35]. Additional research is needed to better understand the association between IPV during pregnancy and healthcare access and use, especially in the postpartum period.

The current study provides a first estimate of the association between IPV during pregnancy on access to family planning in Kenya, determining that experiencing IPV was significantly associated with experiencing issues accessing family planning during COVID-19. An estimated 46% of postpartum women in Kenya report not wanting more children but are not currently using a method of contraception, highlighting the importance of access to contraceptive services [36]. Evidence on the association between IPV and family planning is mixed, with few studies analyzing postpartum contraceptive use specifically. One study from Bihar, India found that physical and sexual IPV were associated with higher odds of modern contraceptive use postpartum [37]. Of 12 global studies included in a systematic review on IPV and contraceptive use, half of the studies reported a positive association, while half reported a negative association between IPV and contraceptive use [16]. In a meta-analysis of 7 of these studies the authors found that, overall, experiencing IPV was associated with lower odds of contraceptive use [16]. An analysis of Demographic and Health Surveys (DHS) from sub-Saharan Africa noted that IPV was positively associated with modern contraceptive use in five of 13 countries [38]. In the 2008 DHS in Kenya specifically, the association was negative but not statistically significant [38]. However, Emenike and colleagues analyzed the 2003 Kenyan DHS, finding that physical, emotional, and sexual IPV were all associated with increased odds of family planning use [39]. Coercion by partners and lack of reproductive autonomy may explain the association between experiencing IPV and experiencing issues accessing family planning found in the current study. Indeed, one mixed-methods study in Nairobi, Kenya identified an association between reproductive coercion and barriers to contraception in a sample of women reporting IPV [39]. Notably, though, studies have focused entirely on the outcome of contraceptive use, emphasizing dynamics in which partners refuse to use contraception [40, 41], rather than exploring more structural factors influencing access to contraception as reported by women in the current study. More research is needed to understand the role IPV may play in structural barriers to accessing postpartum contraception.

This study has several important limitations. First, the study uses a convenience sample of women in two urban counties in Kenya, which may not be representative of the total population of women in Kenya. Second, this study asked about lifetime exposure to IPV. Although a follow-up question provides reasonable certainty that the IPV occurred during pregnancy, it is possible that this assumption of temporality does not hold for all women in the sample. Third, this survey asked women to report on experiences several months in the past, and is potentially affected by recall bias. Fourth, this survey took place during the COVID-19 pandemic, and the findings here may differ from findings outside of the pandemic setting. Still, the findings regarding lack of access to postpartum healthcare and family planning may apply more broadly to times of national emergency. Future studies should examine whether these associations persist in other contexts and settings. Fifth, the overall proportion of participants experiencing issues accessing family planning was small, at just under 10%. This small sample may not be adequately powered to identify the association between IPV and access to family planning. Given that the sample was identified through catchment areas of health facilities, this sample may have greater access to healthcare, including family planning, than the average population. Additional research with larger samples should be conducted to further investigate this outcome. Finally, the multiple choice options for reasons that women avoided postpartum care and specific issued faced in accessing family planning did not include response options related to IPV. Future research should focus on the potential connection between IPV and access to postpartum care, with specific measures capturing this association.

Despite these limitations, this study provides several valuable new findings. Notably, it is the first study to examine the association between IPV and postpartum healthcare. It also provides estimates of the frequency of IPV during the COVID-19 pandemic. Given that Kenya has a high rate of maternal mortality and experienced a surge in violence against women and girls during the COVID-10 pandemic, these findings may be useful for health practitioners and policymakers in Kenya to improve maternal health.

## Conclusion

While many of the measures taken to address the COVID-19 pandemic in Kenya helped decrease the spread of the virus, they may have also increased isolation and household tension, contributing to increased IPV. Although health facilities were still open in Kenya throughout the pandemic, many women may have avoided seeking healthcare due to fear of contracting the virus or lack of funds amid rampant job loss. As such, it is vital that in times of emergency, the government and health facilities emphasize the need for preventative healthcare for postpartum women. In addition to postpartum maternal healthcare, ensuring that women have consistent access to family planning during national emergencies is also vital. Providing timely information about any shortages and supply chain interruptions may help women know where they can go to access family planning. By expecting these service disruptions, healthcare providers should discuss family planning with women during antenatal care and delivery care so they can be prepared after giving birth. Some studies in Kenya have shown that electronic messaging systems may be a useful way to reach mothers to educate them about postpartum healthcare [42] and improve uptake of contraception [43]. Identifying women at risk of IPV before and during pregnancy is also vital. Mathur and colleagues emphasize the need for sexual violence screening [44], while Haberland and colleagues note that in addition to screening, broad counseling on IPV for all women would provide necessary information without requiring women to disclose experiences of IPV [45]. Currently, healthcare providers in Kenya lack training to notice signs of IPV or provide adequate support for women experiencing IPV [33]. Training for healthcare service providers and protocols regarding routine screening and counseling may improve access to care for women experiencing IPV. The Kenyan government and practitioners can learn from the experiences during the COVID-19 pandemic to better prepare for any future emergencies.

## Data Availability

The dataset analyzed during the current study are not publicly available due to ongoing analyses, but are available from the corresponding author on reasonable request.
